# Self-Diffusion in Confined Water: A Comparison between the Dynamics of Supercooled Water in Hydrophobic Carbon Nanotubes and Hydrophilic Porous Silica

**DOI:** 10.3390/ijms232214432

**Published:** 2022-11-20

**Authors:** Michael Fardis, Marina Karagianni, Lydia Gkoura, George Papavassiliou

**Affiliations:** Institute of Nanoscience and Nanotechnology, NCSR Demokritos, 153 10 Aghia Paraskevi, Attiki, Greece

**Keywords:** NMR, QENS, molecule dynamics, supercooled water, carbon nanotubes, MCM-41

## Abstract

Confined liquids are model systems for the study of the metastable supercooled state, especially for bulk water, in which the onset of crystallization below 230 K hinders the application of experimental techniques. Nevertheless, in addition to suppressing crystallization, confinement at the nanoscale drastically alters the properties of water. Evidently, the behavior of confined water depends critically on the nature of the confining environment and the interactions of confined water molecules with the confining matrix. A comparative study of the dynamics of water under hydrophobic and hydrophilic confinement could therefore help to clarify the underlying interactions. As we demonstrate in this work using a few representative results from the relevant literature, the accurate assessment of the translational mobility of water molecules, especially in the supercooled state, can unmistakably distinguish between the hydrophilic and hydrophobic nature of the confining environments. Among the numerous experimental methods currently available, we selected nuclear magnetic resonance (NMR) in a field gradient, which directly measures the macroscopic translational self-diffusion coefficient, and quasi-elastic neutron scattering (QENS), which can determine the microscopic translational dynamics of the water molecules. Dielectric relaxation, which probes the re-orientational degrees of freedom, are also discussed.

## 1. Introduction

The structural and dynamic behaviors of bulk and confined water have been the subject of continuing research due largely to their unusual properties compared with other liquids that are encountered both in their stable and supercooled states [1,2,3,4]. Speedy and Angell, for example, noticed anomalies in the temperature dependence of many thermodynamic response functions and dynamic properties, which were found to diverge at a specific temperature *T*_s_ of around 220 K (at 1 atm) when extrapolated according to a power law function [1]. Unfortunately, when water is cooled at atmospheric pressure, there is a supercooling limit at around 231 K, below which experiments on the liquid phase cannot be performed due to the onset of crystallization to ice [5]. This wide temperature range between the homogeneous nucleation temperature (~235 K) and the crystallization temperature (~150 K) was named ‘no man’s land’ by Mishima and Stanley [6]. Caupin—among others—in his paper [7] surveyed some experiments that attempted to circumvent the above restriction. Water confined in very narrow nanopores was one of these experiments. In this respect, confined water is a subject of continuing interest from both the fundamental and the technological points of view. From the theoretical point of view, confined water is a model system for the study of supercooled water [8]. It is believed that the anomalous behavior of water is best studied at low temperatures within the supercooled region. Additionally, since the homogenous nucleation temperature is a kinetic constraint [5], water molecules’ microscopic dynamics should be of great importance to our understanding of the metastable supercooled phases.

The purpose of this article was not to describe all the work that has been done in the field of bulk and confined stable and supercooled water, nor to summarize the most significant findings. There are many excellent reviews on the structural and dynamic properties of bulk and confined liquids. For a recent review, see, for example, Ref. [9]. Other reviews for confined liquids include Refs. [8,10,11,12,13,14].

The goal of this article was to show, using a particular approach, how it is possible to clearly distinguish between the dynamics of water that is constrained by a hydrophobic and a hydrophilic environment. In this work, no new data are presented. Instead, a specific methodology for analyzing experimental data on the dynamic features of confined water that is available in the literature is described. Intrigued by the extraordinarily fast transport of water in carbon nanotubes (CNTs), we chose these materials as representatives of a smooth hydrophobic surface constraint [15,16,17,18,19]. This fast transport has been attributed to the strong hydrogen bonding between water molecules, which can cause the liquid to recede from nonpolar surfaces to form a vapor layer separating the bulk phase from the surface [15]. On the other hand, cylindrical pores—such as CNTs—but of hydrophilic confinement are excellently represented by the Mobile Composition of Matter No. 41 (MCM-41) material (see, for example, Ref. [8]).

In this work, we will solely concentrate on the assessment of the translational molecular motion of water in the stable and supercooled regimes in both the bulk and the hydrophobic and hydrophilic confined systems mentioned above as determined by the analysis of nuclear magnetic resonance (NMR) and quasi-elastic neutron scattering (QENS) spectroscopic experiments. NMR in a field gradient directly determines the macroscopic translational self-diffusion coefficient *D* without using a model-dependent analysis; QENS can determine the microscopic translational dynamics of water molecules. The inter-relation between the self-diffusion coefficient and the mobility of a system of particles was shown by Einstein, who proved that the diffusion constant *D* of a particle undergoing Brownian motion—typical of a fluid—is related to its mobility *μ* by $D=\mu k_{B}T$, where *μ* is the ratio of the particle’s terminal drift velocity to an applied force. This relationship, known as the Einstein relation, gives us strong experimental evidence to verify that Brownian motion is, in fact, associated with the thermal motion of molecules. This is an example of one of the most general theorems of statistical physics and is called the fluctuation–dissipation theorem [20].

Under this aspect, the unique capability of the NMR gradient technique that measures only the translational motion of the molecules is exploited in this work. However, this technique is only a macroscopic description of the mobility of water being insensitive to the details of the motion; a microscopic one is also needed for a more complete description of the dynamical behavior of the water. 

For typical fluid systems, the microscopic dynamics are usually treated by means of a probabilistic description that uses the ideas of random motions or stochastic fluctuations [21]. The microscopic dynamics are then described only by means of the correlation function of the atomic positions or its Fourier transform, providing the spectral behavior of the random motion. Several spectroscopic techniques have been employed for the study of the random fluctuations in fluid (and solid) systems that differ in the function of the atomic positions, the spectral density of which is detected; and the frequency range, which is possible to explore. Such spectroscopic techniques include, for example, light-scattering experiments, dielectric dispersion and absorption experiments, neutron-scattering experiments, and nuclear magnetic resonance experiments, to mention but a few (see, for example, Refs. [22,23]). 

For instance, in a light-absorption experiment, the spectral density for the autocorrelation function for the electrical dipole moment of the molecule is directly correlated with the line-shape of a rotation–vibration band. In light-scattering experiments, the Raman spectrum is associated with the autocorrelation function for the motion of the polarizability tensor. The Brillouin and Rayleigh scattering spectra can be directly related to the spectral density for the correlation function of time-dependent density fluctuations. In dielectric dispersion and absorption experiments in polar systems, the frequency dependence of the complex dielectric constant allows the relaxation of the electric polarization to be obtained. If a simple exponential form can be assumed for the relaxation function (Debye model), the relaxation time is the correlation time for the orientational fluctuations of the permanent dipole moment. 

Inelastic neutron scattering is a powerful technique that probes a complicated mixture of rotational and translational motion and also is capable of investigating the collective motions characterized by the wave vector *q*. Nuclear magnetic resonance (NMR) relaxation studies in liquids at the Larmor resonant frequency *ω* generally involve complicated expressions that involve the Fourier transform at *ω* of time correlation functions of the dipole field between pairs of diffusing atoms and molecules. In addition to information on the associated correlation times for the motion in liquids, NMR gradient methods, as will be explained in the following, can be conventionally utilized for the measurement of the translational self-diffusion coefficient *D* without requiring a model-dependent analysis. It is this unique capability of this technique that measures only the translational motion of the molecules; being independent of any rotational degrees of freedom, it has replaced the more laborious tracer technique. Dielectric studies, on the other hand are more sensitive to re-orientational degrees of freedom. As it will become clear in the following, NMR gradient measurements of the self-diffusion coefficient of supercooled water below the ‘no-man’s land’were a benchmark for the validity of the subsequent infrared spectroscopic techniques, which were able to indirectly probe the self-diffusion coefficient below the ‘no-man’s land’and provide information for the much-debated state of water. 

As described above, in this work we will provide a detailed analysis of the translational motion of water confined in hydrophobic CNTs and hydrophilic MCM-41 materials as determined by NMR and QENS spectroscopic experiments. Both techniques can yield temporal information about the molecules in fluids and solids with a time scale sensitivity of about 10^0^ to 10^−10^ s for the NMR and 10^−8^ to 10^−14^ s for QENS [24]. Hence, the two techniques complement each other in the window of time. Of course, a comparison of the two techniques mentioned above is not a novel concept. Early studies by Zeidler [25], for example, included comparative studies of QENS and NMR of the molecular motion of liquids, and the advantages and shortcomings of the two methods have been investigated [26]. In addition, Kärger and co-workers extensively used both methods in their studies of confined liquids in zeolites (see, for example, [27]). 

We will show that performing NMR and QENS experiments in the deep supercooled region of water allowed us to clearly observe the differences in the translational dynamics of the bulk and confined systems. It was the combination of these two techniques that could provide a more accurate analysis of the molecular motions of the specific system. More specifically, this procedure involved the accurate determinations of quantities such as the self-diffusion coefficient and the translational correlation times. As a matter of comparison, dielectric measurements, which (in the Debye approximation) probe the rotational degrees of molecular motion, were also included. For the confined systems, we chose to compare a hydrophobic and a hydrophilic one. Despite its long history [28], the phenomenon of hydrophobicity is still under current research [29]; quite recently, the hydrophilic and hydrophobic effects on the structure transport properties of bulk and confined liquid water and its solutions with glycerol and methanol were investigated [30]. 

A rare combination of molecularly smooth walls and hydrophobicity of the surface makes carbon nanotubes a unique model system from both the theoretical and technological points of view. Of course, there are other forms of hydrophobic carbon systems such as carbide-derived carbon [31], carbon nanopores [32,33], and graphite oxide [34]; however, there have been no extensive studies of these materials with a large variety of experimental techniques such those encountered for carbon nanotubes. This is the reason why we focused our attention on the CNT material. Cylindrical pores—such as CNTs—but of hydrophilic confinement are excellently represented by the MCM-41 material. This material has been proven suitable because the silica matrices exhibit regular nanopores of defined and tunable diameters. It has been argued [8] that out of the many hydrophilic porous materials, the water dynamics of MCM-41 (and Santa Barbara Amorphous-15 (SBA-15)) seem to be less influenced by surface interactions, and a more universal relaxation behavior is obtained; thus MCM-41 can be regarded as an ideal model of confined water. Similar to CNTs, a considerable amount of experimental and theoretical work has been carried out on the MCM-41 system. An extensive review of the relaxation behavior of water in a variety of porous materials was presented by Cerveny et al. [8].

Under this aspect, we will show that differences in the dynamic behavior of water in hydrophobic and hydrophilic confinement can be conclusively resolved by performing NMR and QENS experiments in the supercooled state. We will also show that these results were in very good agreement with those obtained in IR experiments using laser heating in thin water films in a deeply supercooled state. Dielectric measurements will also be presented for comparison reasons.

## 2. Bulk Water

### 2.1. Self-Diffusion Coefficient

In general, diffusion is a process controlled by the diffusion equation:

$$\frac{\partial \phi }{\partial t}=D\nabla^{2}\phi$$

where ϕ is a scalar field and *D* is the diffusion constant. The diffusion current according to Fick’s law j=−D∇ϕ relates the flux j of the diffusing species to the gradient of the scalar field [35]. The field ϕ can, for example, be the temperature or relative concentration of two species. In simple fluids, Brownian motion—the random thermal erratic motion exhibited by small particles in suspension in a fluid—is the driving force of the diffusion process and makes the distribution of particles in the fluid tend toward uniformity. According to the diffusion equation, if there is a gradient of the density distribution, a flow is produced, which then induces a change in the density. Hence, diffusion can be considered a macroscopic manifestation of Brownian motion on the microscopic level and is responsible (among the viscous flow and thermal conduction) for the dissipation phenomena that determine the transport properties of a fluid. These dissipative processes are spontaneous microscopic fluctuations that always occur in a system at finite temperatures in the absence of external perturbations [36]. 

Microscopically, the atomic motion of a fluid can be stochastically described by a time-dependent correlation function such as a density-correlation function $G_{s}\left(r,t\right)$ that defines the average density of atoms at the point r at time t if an atom was at the origin r = 0 at time t = 0. Thus, $G\left(r,t\right)$ provides the correlation in the positions of two atoms, which may or may not be different at different times. By closely following March and Tosi [37], contact can be made with the macroscopic diffusional behavior of the fluid by arguing that the self-correlation $G_{s}\left(r,t\right)$—which gives the probability in a one-component fluid of finding the same particle at the position r—must be related to the solution of the diffusion equation because it represents the meanderings of a particle initially at the origin at time t = 0. Now, the diffusion equation is written as:

$$D\nabla^{2}G_{s}\left(r,t\right)=\frac{\partial }{\partial t}G_{s}\left(r,t\right)$$

which implies that the above diffusion equation should be obeyed by $G_{s}\left(r,t\right)$ for times that are long compared with the collision time and for distances that are long compared with the mean free path. Taking into account the initial conditions and probabilistic interpretation of $G_{s}\left(r,t\right)$, the solution of the above equation is:

$$G_{s}\left(r,t\right)={\left(4\pi Dt\right)}^{-\frac{3}{2}}\exp\left(-\frac{r^{2}}{4Dt}\right)$$

Based on phenomenological theory and hydrodynamic arguments, the diffusion coefficient is expected to be related to some mean-square distance over a characteristic time; therefore, the calculation of the mean-square displacement $\langle r^{2}\rangle$ gives:

(1) $$\langle r^{2}\rangle =\int dr\;r^{2}\;G_{s}\left(r,t\right)=6Dt$$

As described above, this result is valid for times that are long compared with the collision times, and it must be expected that $\langle r^{2}\rangle$ will be proportional to t (the slope yielding the diffusion constant).

Therefore, the self-diffusion coefficient D is a time-independent macroscopic hydrodynamic quantity that is governed by the single-particle dynamics of molecules in simple fluids. It is a quantity that can be directly measured in the laboratory by using either single-particle methods or techniques that provide only the averages over particle ensembles [38]. A great number of experiments have been conducted to measure the translational self-diffusion coefficient (D) of bulk water. For early reviews of the results on tracer and self-diffusion coefficients of the various isotopic species of water, see Refs. [39,40]. The NMR technique of spin echoes is conventionally utilized for the measurement of self-diffusion with special variants when the diffusion coefficient is very large or very small [41,42]. The application of a magnetic field gradient over the specimen is central to this method, which encodes the positional information as phase variations. It is important to note that these measurements are purely hydrodynamic in nature; that is, the diffusion coefficients derived are independent of any assumptions concerning microscopic characteristics of atomic motion such as jump length or hard-core diameter; their values are directly related to the lateral molecular displacement [43]. 

**Figure 1:**
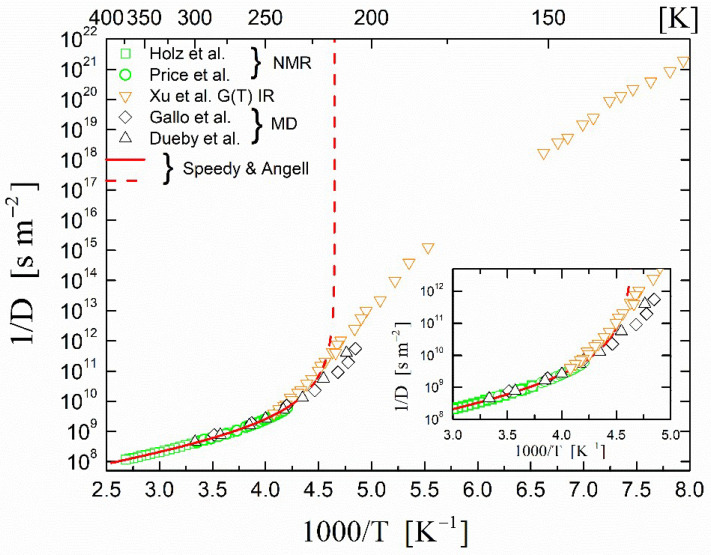
Temperature dependence of the inverse self-diffusion coefficient 1/D in supercooled bulk H_2_O. Squares (**□**) (Holz et al. [44]) and circles (◯) (Price et al. [45]) represent the self-diffusion coefficient (*D*) obtained from pulsed field gradient NMR experiments. The solid line is the singular Speedy and Angell [1] power law fit to the experimental data. The dashed line is the continuation of the fit below 238 K to show the singularity of the power law. Inverted triangles (▽) are self-diffusion coefficients derived from growth rates of crystalline ice experiments (Xu et al. [46]). Diamonds (◊) (Gallo et al. [47]) and upright triangles (△) (Dueby et al. [48]) are self-diffusion coefficients derived from molecular dynamics simulations. The inset is an expanded temperature region around 238 K.

We commence our discussion of the experimental results of D in bulk water H_2_O with two representative sets of ^1^H NMR measurements that employed a pulsed magnetic field gradient: those of Holz, Heil, and Sacco [44] in the temperature range of 273–373 K; and those of Price, Ide, and Arata [45] in the temperature range of 238–298 K. These measurements are shown in Figure 1 in an Arrhenius-type diagram. 

The temperature behavior was clearly of a ‘non-Arrhenius’ nature, a fact that was frequently encountered in the literature and was also applied to other transport properties of water. Many theoretical explanations have been put forward that include, for example, a change in the translational and reorientation dynamics, the coexistence of high- and low-density liquid structures, the increasingly collective character of water motions at low temperatures, the freezing of some collective motions, and a connection of hydrogen-bond exchange dynamics to local structural fluctuations (Ref. [49] and references therein).

Holz et al. [44] found that their data were best fitted with the Speedy–Angell power law [1] given by:

(2) $$D=D_{0}{\left(\frac{T}{T_{s}}-1\right)}^{\gamma },\;$$

where *T*_s_ is the temperature of the thermodynamic singularity and $D_{0}$ and γ are fit parameters. The Speedy–Angell power-law approach is based on the existence of a thermodynamic singularity of water at −45 °C. The solid line in Figure 1 is the fit curve from Holz et al. [44] with the following fitted parameters: $D_{0}$ = 1.635 × 10^−8^ m^2^ s^−1^, $T_{s}$ = 215.05 K, and γ = 2.063. The accuracy of the fit was such that the above authors proposed that the fitted curve could be used for calibration in accurate ^1^H NMR pulsed gradient measurements. The dashed line is an extrapolation of the above fit showing its divergent behavior at $T_{s}$ = 215.05 K. 

The experimental data of Price et al. [45] in the supercooled region can also be fitted with the above power law with the same parameters. Price et al. [45] reported that toward the low-temperature region of their measurements, the diffusion coefficient decreased rather steeply, and at 238 K—the lowest temperature achieved in their experiment—the value of the diffusion coefficient was D = 1.58 × 10^−10^ m^2^ s^−1^. As previously mentioned in the Introduction, below this temperature experiments enter the so-called ‘no man’s land’ due to the onset of ice crystallization. High-quality QENS data obtained by Qvist et al. [50] from bulk water in the range of 253–293 K agreed quantitatively with the NMR results and are not reproduced here.

Computer molecular simulations (MD) have been carried out down to 210 K by many authors in an effort to circumvent the problem of the onset of crystallization. We present here two representative sets of data: those by Gallo et al. [47] and those by Dueby et al. [48]. We observed that the MD data agreed with the NMR data in the high-temperature region but at low temperatures did not diverge toward the ‘discontinuity’ temperature of around 228 K. This was an indication that instead of the presence of singularities in the supercooled region, there might be a continuity of states between the supercooled water and ice phases as proposed by, for example, Lamanna et al. [51].

The continuous change in the thermodynamic properties across the no man’s land was further supported by recent measurements by Xu et al. [46] of the growth rate of crystalline ice and the inference of the diffusivity of supercooled water from 126 to 262 K. They reported the growth rate of crystalline ice *G*(T) using a pulsed-heating technique for 181 ≤ *T* ≤ 262 K and measured isothermally for 126 ≤ *T* ≤ 151 K. Because *G*(T) was proportional to *D*(T), Xu et al. also determined the self-diffusion coefficient *D*(T). These data are shown in Figure 1. The high-temperature data of Xu et al. agreed excellently with those of the NMR measurements as shown in the inset of Figure 1. The non-diverging behavior below 200 K is striking.

We conclude, at this point, our presentation on the dependence of the translational self-diffusion coefficient of bulk water on temperature by noting a quite recent work by Shi, Russo, and Tanaka [52]. These authors proposed a two-state hierarchical model in order to explain the dynamic anomalies of water (see also Ref. [4]). In their paper, among others, they also successfully fit the experimental data of Xu et al. according to this model. A recent publication contained a number of references to the two-state model [53]. 

### 2.2. Correlation Times

Having examined the macroscopic description of liquid water via diffusion experiments, now we turn our attention to what information can be obtained from the microscopic point of view of the system. Contact of this microscopic view with the diffusion process can be made if we consider diffusion as a long time limit of the random flight model in which a molecule suffering a number of collisions executes statistically independent displacements [54]. As was shown in Section 2.1, it is expected that the mean-squared flight distance ($\langle r^{2}\rangle$) and the mean time between flights (τ) are related to the self-diffusion coefficient *D* through relation (1):

$$D=\frac{\langle r^{2}\rangle }{6\tau }$$

In general, the microscopic dynamical behavior can only be described by using the time-correlation function of the atomic positions, which is determined by the thermal fluctuations that occur spontaneously in the equilibrium system [21,22,55]. The correlation function measures the persistence of these microscopic fluctuations, which for a simple fluid-like water are originated by the translational diffusion, rotational tumbling, and molecular vibrations. The correlation function is large at short times and decays to zero at long times; its decay is characterized by the correlation time $\tau_{c}$, a characteristic time scale of the fluctuations determined by the microscopic interactions that is on the order of 10^−12^–10^−13^ sec for simple fluids at room temperature (see, for example, Refs. [23,56]). In this section, we mainly concentrate on the translational and rotational correlation times of the bulk water in its stable and supercooled states.

**Figure 2:**
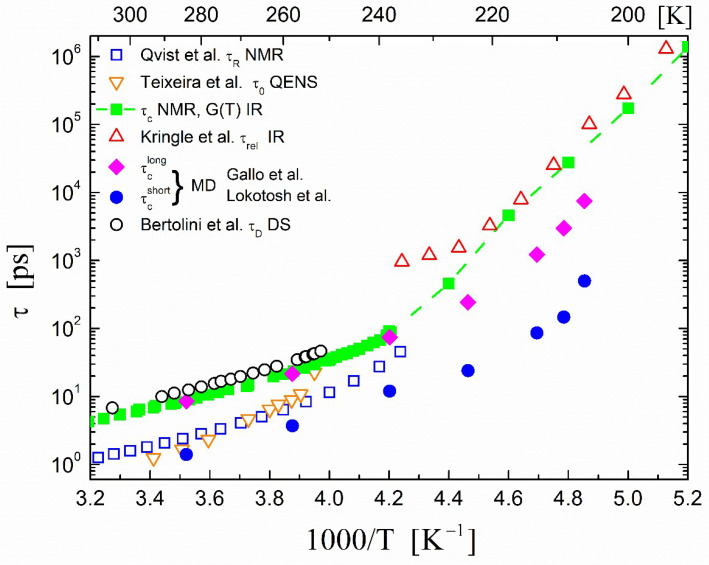
Temperature dependence of correlation times τ in bulk supercooled H_2_O. Open squares (□) are rotational correlation times $\tau_{R}$ derived from ^17^O NMR data (Qvist et al. [67]), inverted triangles (▽) are translational residence times *τ*_0_ obtained from quasi-elastic incoherent neutron-scattering data (QENS) (Teixeira et al. [61]), and the filled green squares (◼) along with the dashed line are the calculated translational correlation times $\tau_{c}$ using Equation (1) (see text). Upright triangles (∆) are the structural relaxation data of pulsed heated water films monitored using reflection-absorption IR spectroscopy (Kringle et al. [59]). The filled circles (●) and filled diamonds (◆) are MD simulations for the correlation times $\tau_{c}^{short}$ and $\tau_{c}^{long}$, respectively, according to Gallo et al. [47] and as presented by Lokotosh et al. [60]. Open circles (◯) are the dielectric (DS) relaxation times $\tau_{D}$ of supercooled water Bertolini et al. [64].

Regarding the NMR method, information on the translational motion in a liquid can in principle be obtained from the T_1_ spin–lattice relaxation time of the hydrogen isotope (see, for example, Ref. [56]). However, in the case of liquid water, the analysis is complicated because apart from the intermolecular relaxation rate (which is characterized by a microscopic correlation time $\tau_{c}$) that eventually determines the translational mobility of the molecules, in water the intramolecular (with a correlation time $\tau_{R}$) and spin–rotational relaxation contributions are also present [57]. Furthermore, it has become evident that within the room temperature range, the correlation times $\tau_{c}$ and $\tau_{R}$ are of equal magnitude. In addition, even in the case that the intermolecular relaxation contribution can be isolated, a value of a hard-core diameter is necessary in order to obtain the translational correlation times from the ^1^H NMR relaxation times. This is usually taken to be equal to the distance of closest approach between the two hydrogen spins (~2.88 Å) and is equated with the rms displacement of a water molecule caused by one translational jump [58]. Hence, we used Equation (1) to calculate the translational correlation times $\tau_{c}$ (using the *D* data of Holz et al. [44], Price et al. [45], and Xu et al. [46]); the results of these three experiments are collectively shown as green squares in Figure 2, in which the dashed line is provided as a guide to the eye. 

The accuracy of using Equation (1) to determine the translational correlation time $\tau_{c}$ from the experimental data of self-diffusion *D* while using an appropriate value (2.88 Å) for the displacement $\langle r^{2}\rangle$ is shown by comparing these results with measurements of $\tau_{c}$ obtained via a different experimental method developed by Kringle et al. [59] within the deep supercooled state. Kringle et al. [59] used infrared (IR) spectroscopy to measure the structural relaxation time of supercooled water for 170 < *T* < 260 K by employing laser pulsed heating; the results are depicted in Figure 2 as red upright triangles. The agreement of the values of $\tau_{c}$ obtained using the above different methods is evident. The results of MD calculations are also shown in Figure 2. We only show the results for correlation times $\tau_{c}^{short}$ and $\tau_{c}^{long}$ obtained by Gallo et al. [47] as presented by Lokotosh et al. [60].

The discussion of the correlation times derived from neutron-scattering studies will now follow. In general, neutron scattering probes a complex mixture of rotational and translational motions in liquids. The two contributions to scattering can, however, be separated in some advantageous cases, which allows information on tumbling motions and diffusional processes to be extracted.

The translational correlation times of supercooled H_2_O derived from high-quality quasi-elastic incoherent neutron-scattering experiments are shown in Figure 2 (Teixeira et al. [61]). The analysis of these particular experimental QENS data assumed a decoupling of rotational and translational motions of the molecules using a random-jump-diffusion model, which provided the translational self-diffusion correlation time D as well as the residence time $\tau_{0}$. The latter residence time was equivalent to the correlation time $\tau_{c}$ measured by the NMR method. However, the resolution of these QENS measurements did not allow for the determination of accurate numerical values of D at low temperatures. Hence, Teixeira et al. used the NMR experimental values of the self-diffusion coefficients obtained from Gillen et al. [62] in the fitting of their data using Equation (1) for the determination of the residence time $\tau_{0}$.

Of equal importance to the translational dynamics are the rotational dynamics of water, particularly the observed coupling of the translational and the rotational motion of the molecules (see, for example, Ref. [63]). 

Dielectric spectroscopy is a valuable tool for the investigation of the dynamics of water. A water molecule possesses a permanent electric dipole moment that couples to external electric fields; in the dielectric experiment, one of the dominant contributions to the dielectric response was the re-orientational time dependence of the electric dipoles. For bulk water, Bertolini et al. [64] measured the dielectric relaxation time of supercooled water at 9.61 GHz; a single Debye-type $\tau_{D}$ was observed down to −18 °C, as shown In Figure 2. The well-known fact that the dielectric relaxation time has the same temperature dependence with the translational correlation time can also be observed in Figure 2.

Nuclear spin relaxation has also been frequently used to study water’s re-orientational motion. One of the most accurate NMR approaches is the determination of the intramolecular rotation correlation time by using ^17^O spin–lattice relaxation measurements that are mainly determined by the rotational local fluctuations of the EFG tensor of the nuclear site within the water molecule (see, for example, Refs. [65,66]).

Qvist et al. [67] reported on such recent ^17^O NMR relaxation data for H_2_O down to 37 K below the equilibrium freezing point. Based on the analysis of the experimental data, they derived the rotational correlation time $\tau_{R}$; the representative results are shown in Figure 2. They also fitted the temperature dependence of $\tau_{R}$ using the singular power law expression:

(3) $$\tau_{R}=\tau_{R0}{\left(\frac{T}{T_{0}}-1\right)}^{-\gamma }$$

They also reported a significant agreement with the previous experimental results of other authors as well as with the MD calculations from their own work. 

The observed differences in the actual values of the rotational correlation times as measured by the ^17^O NMR and the dielectric studies can be attributed to the following fact, which also was observed between the dielectric and depolarized light-scattering experiments [68]. According to Debye’s theory of dielectric relaxation, $\tau_{D}$ is the time in which an assembly of water molecules that is originally oriented by an electric field loses its distribution around a preferred direction due to the Brownian motion after the electric field has been turned off. In the NMR case, according to Bloembergen, $\tau_{c}$ is the time in which a molecule is rotated by the Brownian motion over such an angle that the relative position of the nuclei with respect to the external field and thus the functions of the position’s coordinates—which are functions of spherical harmonics—have changed appreciably [69]. It can be proved that the Debye relaxation time $\tau_{D}$ is related to the correlation time $\tau_{c}$ by the formula $\tau_{D}=3\tau_{c}$. This is because the functions that are used to derive Debye relaxation process are spherical harmonics of order 1, whereas for the NMR correlation times, the spherical harmonics of order 2 are used [70]. However, this factor-of-3 difference between the BDS (l = 1) and NMR (l = 2) correlation times was only found for the limiting case of isotropic rotational diffusion, which according to the simulation results of the Laage group did not apply to the case of water [71]. Another strongly discussed source currently for differences between the results of these methods is that BDS, unlike NMR, also observes cross-correlations between the motions of neighboring molecules.

The relative agreement between the translational and rotational correlation times derived from the NMR and QENS measurements made in these two different ways was most gratifying despite the fact that neutron scattering in general gives slightly smaller values for the translational correlation times [58] in comparison with those obtained from the NMR. The coupling of the translational and rotational degrees of freedom was clearly seen by comparing the QENS experiments with those of the NMR and dielectric ones.

## 3. Confined Water

### 3.1. Self-Diffusion Coefficients

Confined water is a model system for the study of supercooled water [8]. As mentioned in the Introduction, it is a way to avoid the crystallization of bulk water within the no-man’s land. In this work, two sets of measurements of the self-diffusion coefficient of confined H_2_O were considered: those confined in hydrophobic carbon nanotubes and those confined in hydrophilic templated porous silica materials such as MCM-41. Carbon nanotubes (CNTs) have been frequently employed for the study of confined water [15,72]. For the measurements of the self-diffusion of water within hydrophobic carbon nanotubes, we considered those measurements by Mamontov et al. [73], who studied H_2_O confined in single-walled (SW) and double-walled (DW) carbon nanotubes by utilizing high-resolution QENS experiments. In addition, we considered the results of the advanced two-dimensional diffusion–relaxation ^1^H NMR measurements performed by Gkoura et al. [74] in a static magnetic field gradient. For the hydrophilic case, we considered those measurements by Chen et al. [75] and by Weigler et al. [76] of H_2_O in MCM-41 by utilizing NMR experiments. These measurements were compared with the corresponding measurements performed on bulk H_2_O as discussed in Section 2.1. The carbon nanotubes and the MCM-41 materials represented a hydrophobic and a hydrophilic system, respectively. They were chosen among the many confining materials in order to compare the dynamic behavior of water within the two different enclosing environments. The hydrophobicity of water within carbon nanotubes is well documented [15,77]. On the other hand, MCM-41 mesoporous silica can be considered as a prototype hydrophilic matrix for nanoconfinement because it features a regular arrangement of cylindrical pores with controllable uniform diameters and yet a very narrow pore-size distribution [78].

**Figure 3:**
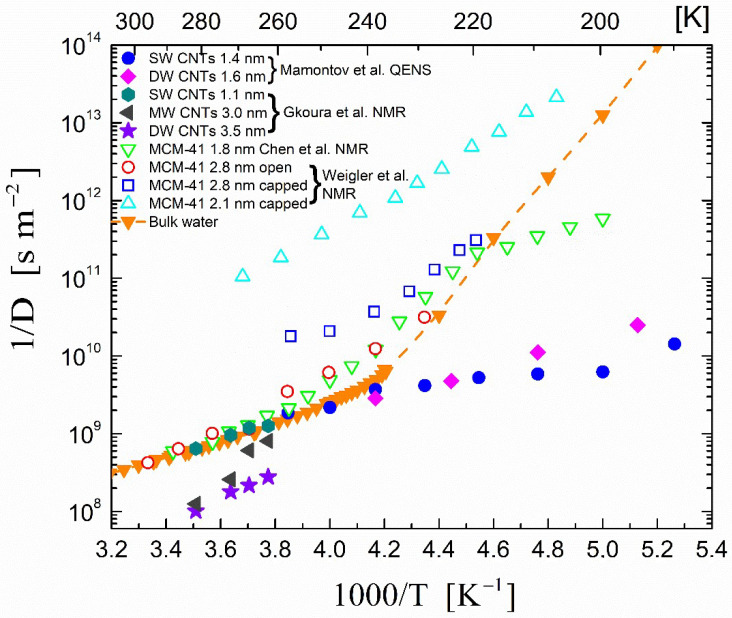
Temperature dependence of the inverse self-diffusion coefficient in supercooled confined H_2_O. The orange line represents a guide to the eye for the available experimental data for bulk H_2_O presented in Section 2.1 (filled inverted triangles (▼)). Filled circles (●) and filled diamonds (◆) represent the self-diffusion coefficient (*D*) obtained from QENS experiments of H_2_O confined in single- and double-walled carbon nanotubes, respectively (Mamontov et al. [73]). Filled hexagons (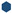), dark grey triangles (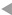), and stars (★) are the *D* values of H_2_O confined in SW 1.1 nm, MW 3.0 nm, and DW 3.5 nm, respectively, acquired via 2D *D*-*T*2 static field gradient NMR experiments (Gkoura et al. [74]). Inverted open triangles (▽) are the self-diffusion coefficients obtained from the pulsed field gradient NMR experiments of water confined in MCM-41 (Chen et al. [75]). Open circles (◯), open squares (□), and open upright triangles (△) are the self-diffusion coefficients obtained from static field gradient NMR experiments on water in open 2.8 nm, capped 2.8 nm, and capped 2.1 nm MCM-41 samples, respectively (Weigler et al. [76]).

The temperature dependence of the inverse self-diffusion coefficient 1/*D* of H_2_O in single-walled (1.4 nm diameter) and double-walled (1.6 nm diameter) carbon nanotubes as obtained from the QENS experiments performed by Mamontov et al. [73] are shown in Figure 3. In the same figure, the 1/*D* values that were obtained from static field gradient NMR experiments performed by our group on SW, DW, and multi-walled MW CNTs of different diameters are also shown. In addition, Figure 3 shows the temperature dependence of the inverse self-diffusion coefficient of H_2_O 1/*D* in MCM-41 as obtained from the pulsed field gradient NMR experiments performed by Chen et al. [75] and the static field gradient NMR experiments of Weigler et al. [76]. 

With regard to the confined water within the carbon nanotubes, it can be observed in Figure 3 that below around 240 K, the self-diffusion coefficients of the confined H_2_O deviated markedly from the corresponding bulk values (filled inverted triangles in Figure 3) and were much larger. Mamontov et al. [73] remarked that the analysis of their QENS data possibly overestimated the values of the diffusion coefficients due to the low-Q data broadening that defined the diffusion coefficients. Nevertheless, higher *D* values at low temperatures for water confined in carbon nanotubes with respect to the bulk values were obtained with other neutron-scattering experiments such as those of Briganti et al. [79]; however, these also suffered from large experimental uncertainty. 

On the other hand, the static field gradient NMR experiments reported in our recent publication [74] also yielded high *D* values for water confined in CNTs, which for a favorable CNT diameter range of 3.0–4.5 nm were found to be higher than the bulk even at temperatures that were not so low. In these experiments, the dynamics of water confined in single-, double- and multi-walled CNTs with different diameters (1.1 to 6 nm) were examined in the temperature range of 265–305 K using two-dimensional NMR diffusion relaxation (*D*-*T*_2eff_) measurements. This method allowed for the experimental identification of distinct water groups with characteristic diffusion and relaxation profiles and confirmed the predictions of molecular dynamics simulations that visualized a stratified arrangement of water inside the nanotubes. A single *D* component was found for water inside the narrow (1.1 nm) SW CNTs, whereas for CNTs with diameters in the range of 3.0–4.5 nm, the nanotube water was shown to organize into two concentric tubular sheets with distinct *D* values and with the *D* values of the central axial component being almost 4 times larger than in the bulk. The 1/*D* values of the nanotube water inside the SW 1.1 nm CNTs and of the central axial water component in the MW 3.0 nm and DW 3.5 nm CNTs are depicted in Figure 3. For the central axial water component, the extremely fragile liquid behavior as indicated by the markedly non-Arrhenius temperature dependence of the diffusion coefficient should also be noticed in Figure 3. At this point, it should be underlined that several studies [19,80,81,82,83,84,85] identified a certain width—critical diameter (d < 1.0 nm)—up to which water diffused in a single-file mechanism, while for larger (d > 1.0 nm) CNT diameters [15,86,87], a layered water structure appeared in accordance with the studies of our group [74,88]. By further increasing the CNT diameter, the diffusion characteristics of the internal water molecules mainly located at the center of the tube approached those of the bulk liquid.

Regarding water confined inside hydrophilic nanopores, the inverse of the self-diffusion coefficients 1/*D* of water measured via pulsed gradient NMR as a function of 1/*T* for a fully hydrated MCM-41-S sample (micelle-templated nanoporous silica matrix) with a pore diameter of 1.8 nm (experiments with a 1.4 nm pore diameter have shown the same results) are shown [75] in Figure 3. In addition, in a quite recent work, Weigler et al. [76] performed static field gradient NMR experiments on water confined in open and capped MCM-41 samples with pore diameters ranging from 5.4 nm to 2.1 nm. In Figure 3, only the results of the 2.8 and 2.1 nm pore diameters are presented for clarity. 

As evidenced in Figure 3, in contrast to the water confined in the carbon nanotubes, the diffusion coefficient *D* of H_2_O confined in MCM-41 had lower values compared to those of the bulk water. The only exception to this general trend was the observation of a crossover in the self-diffusion data of the hydrated MCM-41-S sample at around 225 K, as seen in Figure 3. This was proposed to be due to a dynamical crossover from a fragile to a strong behavior of water found via computer simulations and experiments, as described for example in a recent review by De Marzio et al. [89]. However, this interpretation is highly debated; it has been argued that this crossover phenomenon is not a true fragile-to-strong transition but rather is due to the vanishing of the cooperative α relaxation [90,91] or to a crossover from bulk-like to interface-dominated dynamics [92]. For a detailed discussion of this dynamic crossover under confinement, see, for example, Ref. [8]. It is noteworthy that at this crossover temperature, the values of the confined 1/*D* coefficients coincide with those of the bulk water, as seen in Figure 3. Whether or not this was a fortuitous observation, other experimental observations of self-diffusion coefficients in confined geometries should be considered.

### 3.2. Correlation Times

**Figure 4:**
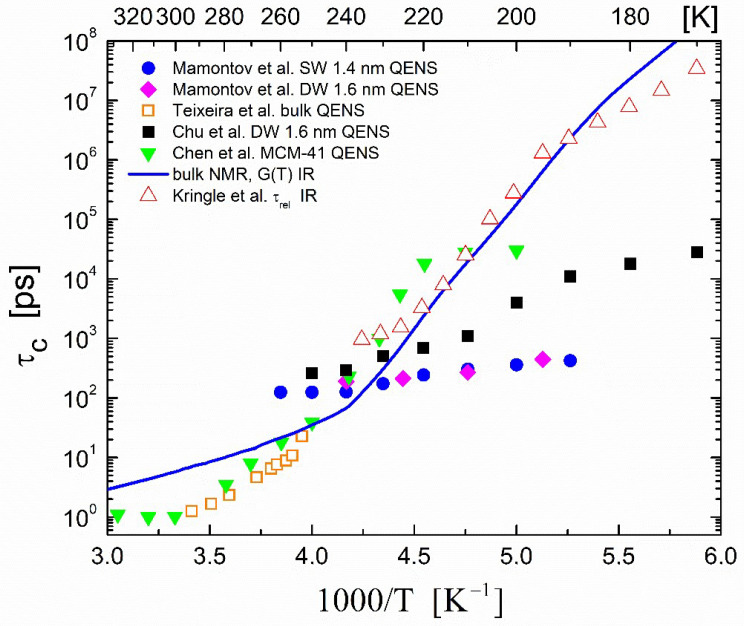
Temperature dependence of the translational correlation times (residence times in QENS experiments) in supercooled confined H_2_O. Filled circles (●) and filled diamonds (◆) represent the residence times $\tau_{0}$ obtained from QENS experiments of H_2_O confined in single-walled (1.4 nm) and double-walled (1.6 nm) carbon nanotubes, respectively (Mamontov et al. [73]). Filled squares (◼) represent the residence times $\tau_{0}$ obtained from QENS experiments of H_2_O confined in 1.6 nm double-walled carbon nanotubes Chu et al. [93]. Inverted filled triangles (▼) are the residence times obtained from QENS experiments of H_2_O confined in MCM-41-S samples with a 1.8 nm pore diameter (Chen et al. [75]). Orange open squares (**□**) represent the residence times $\tau_{0}$ obtained from QENS experiments of bulk H_2_O (Teixeira et al. [61]). The blue solid line is a guide to the eye for the calculated correlation times obtained from the NMR experiments by Holz et al. [44] and Price et al. [45] and for the growth rate from pulsed heating experiments by Xu et al. [46]. Upright triangles (∆) are the structural relaxation data of pulsed heated water monitored via reflection–absorption IR spectroscopy (Kringle et al. [59]).

Compared with the experimental uncertainty of the *D* measurements of the confined water in carbon nanotubes in the QENS experiments of Mamontov et al. [73] discussed in Section 3.1, the reliability of the derived residence times $\tau_{0}$—equivalent to the translational correlation times—are much higher because they are determined using the high-Q data, therefore providing reliable information on the translational diffusion process. The temperature dependencies of the residence times $\tau_{0}$ for the supercooled water within the single-walled (1.4 nm) and double-walled (1.6 nm) carbon nanotubes are shown in Figure 4. 

Two sets of QENS measurements of supercooled H_2_O confined in carbon nanotubes are presented in Figure 4: those by Mamontov et al. [73] for single-walled (1.4 nm) and double-walled (1.6 nm) carbon nanotubes and those of Chu et al. [93] for 1.6 nm double-walled carbon nanotubes. For the hydrophilic case, the translational correlation time obtained from the QENS spectra for water confined in the MCM-41-S sample with a pore diameter of 1.4 nm (Chen et al. [75]) is shown in Figure 4. This group reported similar values of the correlation times for a 1.8 nm sample. The temperature dependencies of these correlation times were quite different from those obtained for the bulk supercooled water. 

For this bulk water, the correlation times obtained via NMR, QENS, and IR are shown in Figure 4. The blue solid line is a guide to the eye for the correlation times obtained from the pulsed gradient NMR experiments by Holz et al. [44], Price et al. [45], and Xu et al. [46] by using Equation (1) as described in Section 2.2. The QENS data were from Texeira et al. [61], and the structural relaxation data of the pulsed heated water monitored via reflection–absorption IR spectroscopy were from Kringle et al. [59].

We observed that below around 210 K, all of the translational correlation times for the confined supercooled water in the carbon nanotubes remained lower than the corresponding ones for the bulk supercooled water, which indicated a faster translational motion within the tubes. This was in accordance with the diffusion data presented in Figure 2. Therefore, water in supercooled carbon nanotubes attained a higher mobility than the bulk supercooled water.

In MCM-41, however, the confined water had a completely different behavior with respect to the bulk one, as shown in both Figure 3 and Figure 4. As shown in Figure 3, the self-diffusion coefficients *D* of the confined water in MCM-41 as obtained from pulsed field gradient NMR experiments had distinctly lower values compared to those of the bulk ones. In addition, as shown in Figure 4, the correlation times for the MCM-41 case as obtained from the QENS experiment had values that were higher or very close to those of the bulk water. Thus, the experiments indicated that the confined water molecules within the MCM-41 attained lower or similar translational mobilities with respect to the bulk water molecules.

**Figure 5:**
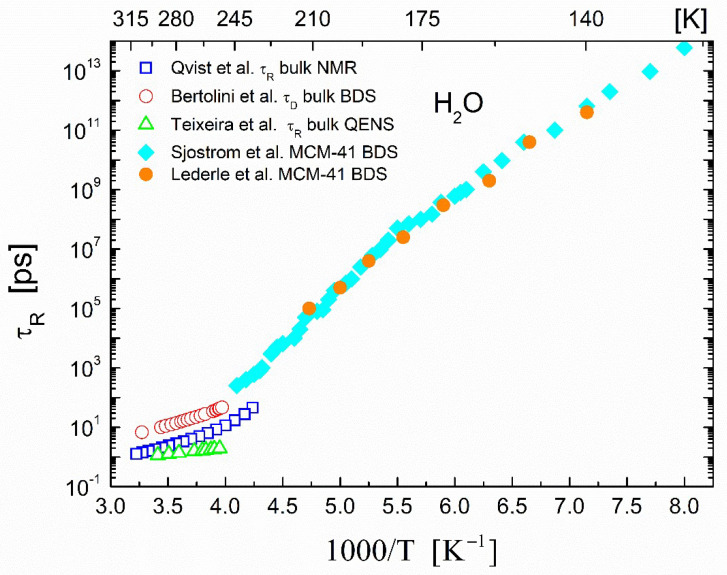
Temperature dependence of rotational correlation times $\tau_{R}$ in bulk and confined water H_2_O. Open squares (□) are the rotational correlation times $\tau_{R}$ of bulk H_2_O obtained from ^17^O NMR experiments (Qvist et al. [67]). Open circles (◯) are the corresponding $\tau_{D}$ values reported from the dielectric measurements of Bertolini et al. [64]; open upright triangles (△) are the $\tau_{R}$ values in bulk H_2_O obtained from the QENS experiments of Teixeira et al. [61]. Filled diamonds (◆) are the correlation times $\tau_{R}$ for supercooled H_2_O within MCM-41 pores with a diameter of around 2.1 nm obtained from the broadband dielectric spectroscopy (BDS) measurements of Sjöström et al. [94]. Filled circles (●) are the corresponding $\tau_{R}$ values for supercooled H_2_O confined in MCM-41 pores with a 2.5 nm diameter reported by Lederle et al. [92].

Finally, we present a representative set of measurements of the rotational correlation times $\tau_{R}$ obtained by the three different experimental methods (NMR, QENS, and dielectric) discussed in Section 2.2. As mentioned in Section 2.2, a considerable number of NMR measurements have been taken for the determination of the rotational correlation time in water using the ^17^O isotope. In this case, the rotational motion of individual molecules was detected through the interaction of the nuclear electric quadrupole with the intramolecular electric field gradient at the nuclear site. The $\tau_{R}$ times from the ^17^O spin–lattice relaxation NMR measurements by Qvist et al. [67] of ^17^O-enriched bulk water are shown in Figure 5. In addition, alongside the bulk water $\tau_{R}$, Figure 5 shows the results of two sets of dielectric measurements on water confined in MCM-41: those of Sjöström et al. [94] in MCM-41 with a pore diameter of around 2.1 nm and those of Lederle et al. [92] with an MCM-41 pore diameter of around 2.5 nm. For the confined water, multiple relaxation processes were identified in both sets of measurements [92,94]. Here, we reproduced in Figure 5 only the results for the relaxation process that was assigned to supercooled water. This figure exemplifies the best manner in which confined systems are model systems for the study of the dynamics in bulk water and that in the particular system, only the broadband dielectric relaxation studies could penetrate deep into the supercooled region beyond the ‘no-man’s land’ and measure the rotational correlation time of the water molecules. 

**Figure 6:**
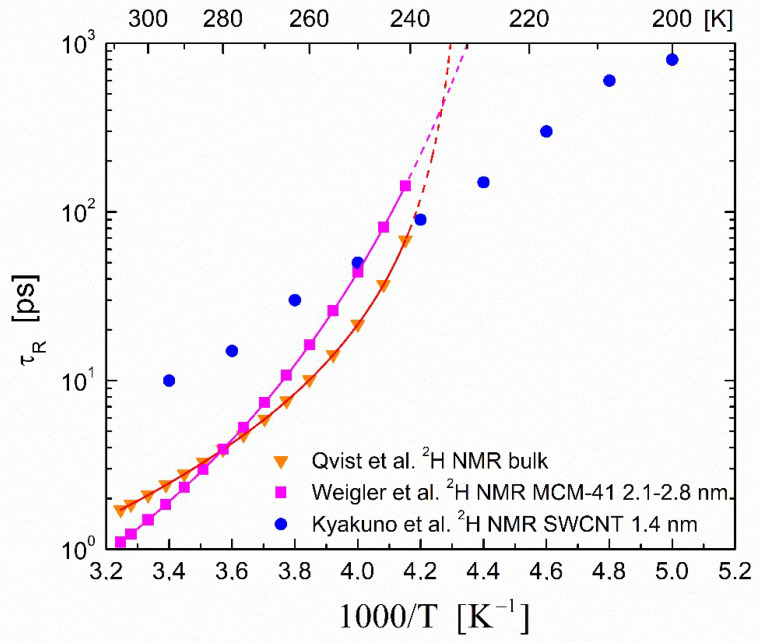
Temperature dependence of the rotational correlation times $\tau_{R}$ in bulk and confined heavy water D_2_O. Inverted triangles (▼) are the rotational correlation times $\tau_{R}$ of bulk D_2_O obtained from ^2^H NMR experiments (Qvist et al. [67]). Filled circles (●) are the correlation times for D_2_O within 1.4 nm SW carbon nanotubes obtained from ^2^H T_1_ NMR data (Kyakuno et al. [95]). Filled squares (◼) are the rotational correlation times for D_2_O confined in MCM-41 with pores in the range of 2.1–2.8 nm obtained from ^2^H T_1_ and stimulated-echo experiments (Weigler et al. [96]).

Alternatively, a commonly employed method for determining rotational correlation times is to perform ^2^H NMR measurements in heavy water using the ^2^H isotope as a probe. The representative correlation times $\tau_{R}$ derived from ^2^H NMR measurements in bulk and confined heavy water D_2_O are shown in Figure 6. 

For bulk heavy water, we present selected $\tau_{R}$ data from recent measurements by Qvist et al. [67]. The line is a power law fit to Equation (3) with parameters given in the work of Qvist et al. [67]. The dashed line is an extrapolation that shows the diverging behavior of the power law. 

For the confined heavy-water systems, we chose to present the most recent results of Weigler et al. [96] on the reorientation of deeply cooled water in mesoporous silica. Weigler et al. [96] obtained the correlation times $\tau_{R}$ from ^2^H spin–lattice relaxation and stimulated-echo experiments on D_2_O confined in the mesoporous silica MCM-41 and SBA-15 with various pore diameters. The results for the MCM-41 system with pores in the range of 2.1–2.8 nm are presented in Figure 6. These data were fitted by a Vogel–Fulcher–Tamman expression (shown as solid line in Figure 6); the dashed line is an extrapolation that shows the divergent behavior of the fit. As can be seen in the data above T~240 K, the rotational correlation times for the confined water were in reasonable agreement with the ones for the bulk water. Weigler et al. [96] reported that the above statement was also true for the low-temperature data in the deeply cooled state (not shown; details can be found in their work).

In contrast, the rotational correlation times $\tau_{R}$ of heavy water molecules inside SWCNTs with mean diameters between 1.45 and 4 nm reported by Kyakuno et al. [95] and obtained via ^2^H NMR exhibited an entirely different behavior, as can be seen in Figure 6 for the case of 1.45 nm. In the weakly cooled temperature range above ~240 K, the confined system exhibited slower dynamics (increased $\tau_{R}$) compared with the corresponding bulk case, presumably due to a fast exchange between the adsorbed and central bulk-like molecules within the CNT matrix. However, it was found that in the deeply cooled temperature range, the water molecules exhibited faster dynamics compared to the bulk molecules. Kyakuno et al. [95] also compared their data with MCM-41 literature data (Figure 5 in their work) and reported that these faster water dynamics could be achieved by increasing the hydrophobicity of the pore walls and decreasing the pore diameters.

In conclusion, the general behavior was that the measured self-diffusion coefficients of H_2_O confined in MCM-41 or in carbon nanotubes were of lower or higher values, respectively, compared to those of the bulk water in the supercooled region. The same behavior was observed for the mobilities of the confined water as measured by the translational correlation times as well as by the measured rotational dynamics of water molecules. We observed lower correlation times (higher mobility) for water confined in carbon nanotubes with respect to the bulk values in the deep supercooled state. In contrast, water confined within MCM-41 exhibited longer or similar correlation times compared to the bulk water within the entire temperature range examined. These differences can be ascribed to the hydrophilicity of the MCM-41 as compared to the hydrophobicity of carbon nanotubes. 

Another exemplary system in which hydrophobic–hydrophilic interactions play an important role is the three-component nanocomplexes comprising CNTs—ionic surfactants—and π-conjugated organic dyes [97,98,99]. In these systems, the hydrophobic/hydrophilic interaction of the surfactant with the CNT/dye, respectively, is believed to promote the formation of resonantly coherent J-aggregates on the external surfaces of the CNTs, which leads to highly efficient resonant energy transfer from the dye to the CNT. Studies of the diffusion dynamics in these systems using the procedure outlined above would be extremely valuable in elucidating the exact role of the surfactant and the dye interaction on the aggregation process. 

It should be stressed that apart from the self-diffusion NMR data between 238 and 373 K, the self-diffusion coefficients and the translational and rotational correlation times presented were experimentally obtained via different techniques. The difference in the dynamic behavior of the confined water in the two different confinements examined was observed in both the self-diffusion and correlation time experiments. This outcome was consistent with studies that compared the structure and dynamics of liquid water on hydrophobic and hydrophilic surfaces using a molecular dynamics simulation [100]. In that study in particular, it was found that the calculated diffusion coefficients of water in the first layers near the hydrophilic surface were lower than those in the first layers of the hydrophobic surfaces. 

It should be mentioned that all in general, molecular simulation studies concerning the dynamics of water in confined geometry are conducted at temperatures below around 230 K—as, for example, in Ref. [101]—where the differences in the self-diffusion coefficients between bulk and confined water are rather small. In this article, we showed that in the supercooled temperature region, these differences in the self-diffusion coefficients and the correlation times between the hydrophobic and hydrophilic confinements were beyond experimental uncertainty. 

## 4. Conclusions

In this work, we showed that the combined experimental QENS and NMR study of water confined in hydrophobic and hydrophilic nanopores can explicitly distinguish hydrophobic from hydrophilic environments in the supercooled temperature region based on the values and the temperature dependence of the self-diffusion coefficients and the translational correlation times of the confined water. In particular, it was shown that in the hydrophobic case, the water dynamics exhibited a highly fragile liquid behavior with *D* values markedly larger and correlation times shorter than those in the bulk. On the contrary, the *D* values for water in hydrophilic nanoconfinement were found to be considerably lower and the translational correlation times much longer than the corresponding bulk values. This was demonstrated by comparing the representative results acquired via both experimental methods of QENS and NMR diffusion spectroscopy. The results for the bulk water were in very good agreement with those obtained by IR spectroscopy in laser heating experiments on water films in the deep supercooled state. It would be very beneficial to extend these IR laser heating experiments to confined water.

The present work provided strong evidence that the proposed methodology in the analysis of the combined experimental techniques can help to elucidate the nature and properties of the interaction between water molecules and the confining matrix. Understanding the water-confining matrix interactions and how they influence the behavior of water under nanoconfinement is a crucial step toward gaining a deeper insight into many intriguing water properties and clarifying theoretical predictions in domains inaccessible in the bulk phase.

## Data Availability

The data that support the findings of this study are available from the corresponding author upon reasonable request.
